# Construction and Testing of *orfA* +/- FIV Reporter Viruses

**DOI:** 10.3390/v4010184

**Published:** 2012-01-23

**Authors:** Hind J. Fadel, Dyana T. Saenz, Eric M. Poeschla

**Affiliations:** 1 Mayo Clinic, Department of Molecular Medicine, Guggenheim 18-11A, College of Medicine, 200 First Street SW, Rochester, MN 55905, USA; Email: saenz.dyana@mayo.edu; 2 Mayo Clinic, Division of Infectious Diseases, Guggenheim 18-11A, College of Medicine, 200 First Street SW, Rochester, MN 55905, USA; Email: fadel.hind@mayo.edu

**Keywords:** FIV, lentivirus, reporter virus, OrfA

## Abstract

Single cycle reporter viruses that preserve the majority of the HIV-1 genome, long terminal repeat-promoted transcription and Rev-dependent structural protein expression are useful for investigating the viral life cycle. Reporter viruses that encode the viral proteins *in cis* in this way have been lacking for feline immunodeficiency virus (FIV), where the field has used genetically minimized transfer vectors with viral proteins supplied *in trans*. Here we report construction and use of a panel of single cycle FIV reporter viruses that express fluorescent protein markers. The viruses can be produced to high titer using human cell transfection and can transduce diverse target cells. To illustrate utility, we tested versions that are (+) and (-) for OrfA, an FIV accessory protein required for replication in primary lymphocytes and previously implicated in down-regulation of the primary FIV entry receptor CD134. We observed CD134 down-regulation after infection with or without OrfA, and equivalent virion production as well. These results suggest a role for FIV proteins besides Env or OrfA in CD134 down-regulation.

## 1. Introduction

Initial analyses of a retrovirus typically involve monitoring productive replication of clinical isolates or passaged viral strains. To analyze the viral replication process more precisely, genetically defined full-length infectious molecular clones are utilized. However, proper interrogation of specific life cycle steps — entry, reverse transcription, nuclear import, integration, assembly, etc. — typically requires a replication-defective system in which single infection cycles can be assessed quantitatively and viral production (late events) can be separated experimentally from the infection events spanning entry to integration (early events). Reporter genes that can be monitored more readily than viral antigens can be inserted and ease of containment may be enhanced. Two types of retroviral vector systems are used for this purpose. One characteristically expresses most retroviral proteins *in cis* (reporter viruses) and the other *in trans* (typical gene therapy vectors). Each has specific advantages that depend upon the experimental goal. In the case of HIV-1, split-component systems in which the genome is a minimal transfer vector are readily available for basic and translational research but are preferred in the gene therapy setting [1]. For basic virology in which the goal is to analyze the life cycle with fidelity to the natural situation, it is often more desirable to use an HIV-1 reporter virus that has been rendered minimally replication-defective with a frame-shift or deletion in *env*. This is particularly advantageous if the complex transcription, genomic RNA splicing and protein expression cascade that eventuates in Rev-dependent protein accumulation is under study. Either HIV-1 Env or a different envelope protein capable of pseudotyping is provided *in trans*. Since the viral *nef* gene is dispensable for most studies of HIV-1 in cultured cells, its open reading frame has traditionally provided an optimal locus for inserting a luciferase or GFP cDNA [2,3,4]. HIV-1 reporter viruses of this kind have facilitated a large number of basic research studies in the past two decades.

Non-primate lentiviruses such as FIV, EIAV and Visna provide informative comparative models, and in the case of FIV, AIDS can be studied. For these lentiviruses, minimal vector systems have been engineered [5]. FIV vectors have recently seen markedly increased use in basic virology studies conducted by HIV investigators, most prominently for investigation of species-specific post-entry restriction mechanisms [6,7,8,9,10,11,12,13,14,15,16,17,18,19,20,21,22,23,24,25,26,27,28,29,30,31]. However, reporter viruses analogous to those available for HIV-1 have been lacking. One main reason is that an optimal approach for marker gene insertion into a full-length or *env*-minus FIV genome is less evident than it is for the primate lentiviruses. FIV lacks a *nef gene* or other accessory gene-encoding open reading frame similarly situated at the 3’ end of the genome. Here we describe the construction and initial use of FIV reporter viruses.

## 2. Results

**Figure 1:**
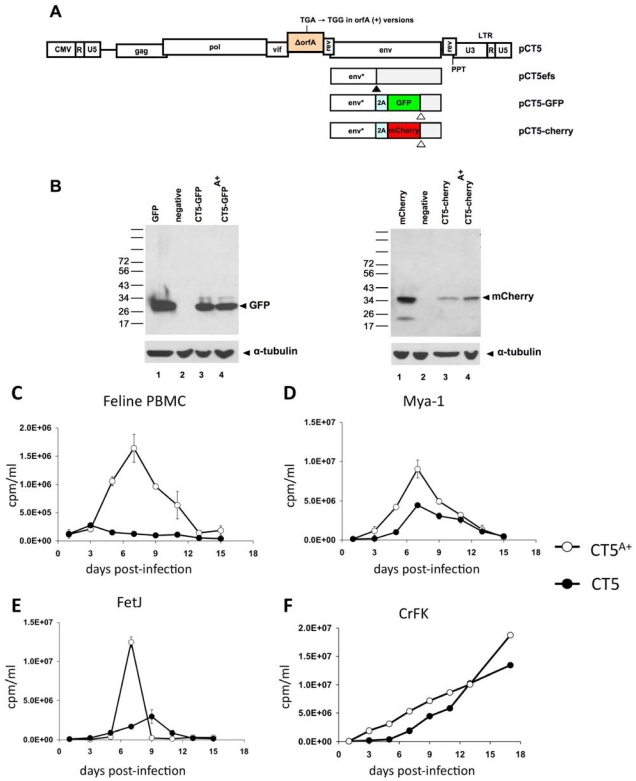
**Virus design and testing of biological activity of restored OrfA in replicating FIV**. (A) Virus genome arrangements. U3: 3’ unique element. U5: 5’ unique element. R: repeat element. PPT: polypurine tract. 2A: porcine teschovirus 2A peptide. CMV: human cytomegalovirus immediate early gene promoter. efs: envelope frame shift (black arrowhead). CT5efs has a frameshift in *env*, which was used as the insertion point for the 2A peptide as well. Env*: N-terminal Env protein fragment resulting from the frame shift. Open arrowheads indicate the GFP and mCherry stop codons (the *env* mRNA fragment distal to the stop codons is untranslated). (B) Immunoblotting demonstrates that the P2A peptide results in co-translational cleavage and generation of free GPF (left) and mCherry (right). Lane 1: cells expressing eGFP or mCherry; Lane 2: untransfected negative control; Lanes 3 and 4: reporter viruses. 293T cell lysates were harvested 48 hours after transfection and blotted with antibodies to GFP or mCherry. (C-F) Replication of viruses produced from CT5 and CT5^A+ ^in primary feline PBMC, feline T-cell lines Mya-1 and FetJ, and CrFK cells. Cells were not induced with any additional agents, such as soluble CD134 [34] with the exception that Mya-1 cells were maintained with human IL-2 as described in Section 3. Error bars represent standard deviation of duplicate measurements.

The virus design strategy needed to take into account two main considerations. First, highly transfectable feline cell lines conducive to high titer virus production are not available (i.e., no feline equivalent to commonly employed 293T or COS producer cells). Second, there are several operationally important differences between the genomic organization of this lentivirus and primate lentiviruses. The viruses we constructed here (Figure 1A) are based on the previously reported pCT5 modification [5,32] of the FIV 34TF10 molecular clone [33]. In the parental plasmid pCT5, the essential modification of the FIV genome is that the promoter-containing 5’ U3 element is replaced by the human cytomegalovirus immediate early gene (hCMV) promoter. The fusion is at the TATA box located just upstream of the 5’ R repeat [5,32] (Figure 1A). Because the FIV U3 displays minimal promoter activity in human cells, this modification permits genetically defined FIV genomes and their encoded proteins to be produced at high yield by transfection of well-characterized human cell lines, e.g., 293T cells [5,32]. The other main genomic organization constraint to be circumvented is that no open reading frame analogous to *nef* exists in FIV. Additionally in contrast to the primate lentiviruses, the indispensable second exon of Rev overlaps with the polypurine tract and the 3’ U3 element. Therefore, the 3’ end of the FIV genome does not provide a natural insertion point for a marker gene. 

To address this issue, we instead expressed the marker proteins eGFP or mCherry as 2A peptide-linked in-frame insertions in the central region of *env* (Figure 1A). 2A peptides, originally identified in foot-and-mouth disease virus [35], are 18-22 amino acid peptides that contain a conserved Asp-Val/Ile-Glu-X-Asn-Pro-Gly°Pro motif [36]. This extremely rare sequence mediates ribosomal skipping and co-translational cleavage between the terminal Gly and Pro (arrow, previous sentence) without affecting translation of the downstream (“2B”) portion of the polypeptide. Here the 19 amino acid porcine teschovirus-1 2A (P2A) peptide was used and inserted such that a segment encoding 152 N-terminal amino acids of FIV SU are upstream. This strategy avoids intrusion of the marker gene upon either exon of Rev or the RRE, and it moreover preserves the *vif* and *orfA* genes for experimental analysis. Microscopy showed that cells transduced with these viruses were homogeneously fluorescent for GFP or mCherry (data not shown). Consistent with this, immunoblotting showed that the P2A peptide resulted in production of free fluorescent proteins (Figure 1B). We then created OrfA (+) and (-) versions of each of the eGFP and mCherry reporter viruses. We did so by first repairing in pCT5 the premature stop codon retained from the parental provirus. The OrfA (+) version pCT5 is termed pCT5^A+^. 

**Figure 2:**
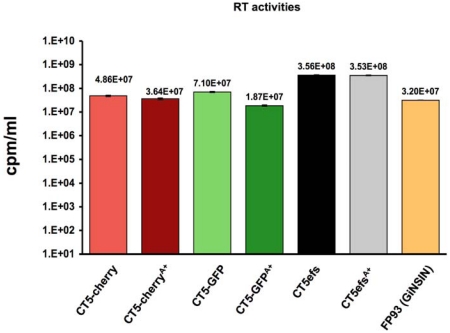
**Virus production**. Supernatant reverse transcriptase activities of the different reporter viruses. For vector GiNSIN, the source of RT activity is the packaging plasmid pFP93 used to trans-package the transfer vector [6,40]. Background RT activities determined in supernatants of un-transfected control cells, which were between 0.1 - 1 % of transfected cell supernatant values, were subtracted before graphing. Error bars represent standard deviation of duplicate measurements.

*orfA* is required for FIV replication in feline PBMCs and some T cell lines but not CrFK cells [37,38,39]. To verify that the *orfA* allele we generated by conversion of the premature stop codon to a tryptophan codon is functional, primary feline PBMCs, the feline T-cell lines FeT-J and Mya-1, and CrFK cells were infected with pCT5 and pCT5^A+^-generated virus particles at equivalent MOI. The greatest disparity was observed in PBMC, where the CT5-generated virus did not replicate productively while CT5^A+^ did so robustly (Figure 1C). In the two lymphoid cell lines the CT5^A+^ virus achieved higher peak levels (Figure 1D,E). These results confirm the biological activity of the repaired *orfA* (+) allele and previous reports that it is required for replication in feline PBMC.

The active *orfA* allele was then incorporated into the reporter viruses by exchange of restriction fragments containing the gene. Supernatant RT activities produced by transient transfection of pCT5, pCT5-cherry, pCT5-GFP and the respective OrfA+ versions were equivalent (Figure 2) and at 10^7^ to more than 10^8^ RT units per ml are also comparable to those produced by FIV packaging plasmid pFP93 [6,40]. Thus, the marker gene insertions did not detectably impair late events from transcription through splicing, Rev-dependent protein production and budding, and OrfA was neutral in this regard as well.

**Figure 3:**
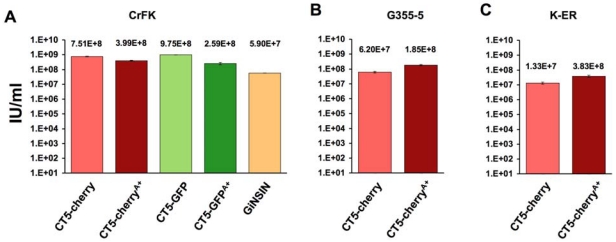
**Reporter virus titers on (A) CrFK; (B) G355-5; and(C) FetJ cells**. Viruses and the GiNSIN vector were pseudotyped here and in following experiments with vesicular stomatitis virus glycoprotein G (VSV-G). Error bars represent standard deviations of duplicate measurements.

Reporter virus titers were then determined by flow cytometry for GFP or mCherry (Figure 3A-C). They ranged between 10^7^ and 10^9^ transducing units per ml in several feline cell lines and were equivalent to standard FIV vectors generated with pFP93 and the minimal FIV transfer vector GiNSIN [40,41,42,43]. 

**Figure 4:**
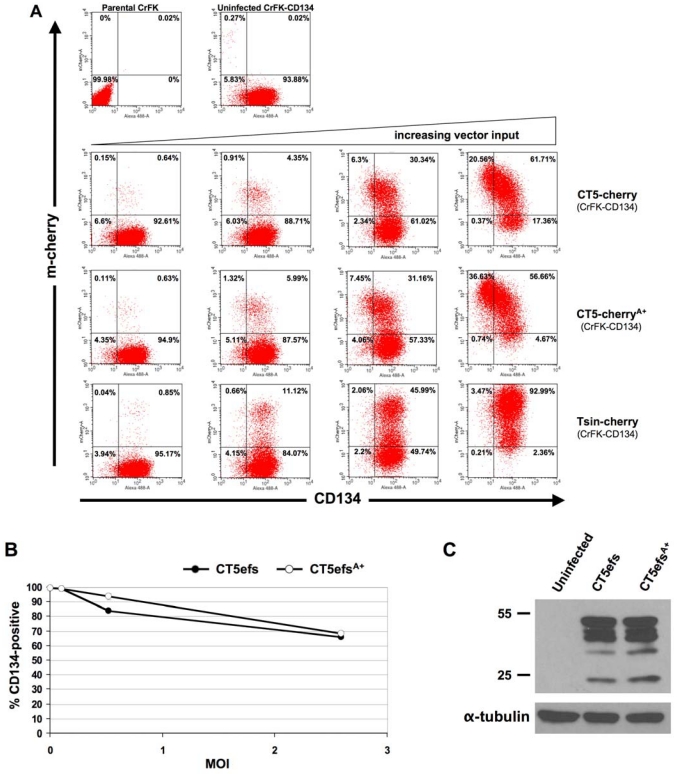
**Testing of OrfA (+) and (-) reporter virus.** (A) CrFK-CD134 cells were derived as described in the Experimental Section and the stable cell line was infected with increasing amounts of each CT5cherry or CT5-cherry^A+^ reporter virus. Lentiviral vector TsinCherry [25], which expresses mCherry under control of the hCMV promoter but encodes no viral proteins, was used as a control. Flow cytometry for CD134 surface expression was performed at 72 hours. (B) 30,000 CrFK-CD134 cells were infected with CT5efs and CTefs^A+^ viruses at equivalent MOI (0.5 and 2.6). MOI was calculated by pre-titration on CrFK-CD134 cells with the focal infectivity assay of Remington et al. [50]. Flow cytometry for CD134 surface expression was performed at 72 hours. Error bars represent standard deviations of duplicate measurements. (C) Immunoblotting for FIV Gag/Pol (top blot) and tubulin (bottom blot) in lysates of the cells shown in panel B (MOI = 2.6). Numbers indicate MW markers (kDa).

Multiple viral life cycle functions have been provisionally assigned to OrfA in previous studies, including a promoter transactivation role analogous to that of the HIV-1 Tat protein, cell cycle arrest induction, protein ubiquitination, CD134 down-regulation and a role in virus assembly and particle formation [39,44,45,46,47,48,49]. Most recently, Hong et al. reported that OrfA mediates CD134 down-regulation and is required for FIV 34TF10 replication in CrFK cells that express CD134 (GFox cells) [46]. To initiate our own analyses of the role of this viral accessory protein, we independently generated CrFK cells that express feline CD134 (Figure 4). 

We then infected them with serial dilutions of RT activity-normalized VSV-G pseudotyped reporter viruses. At 72 hours, cells were examined by flow cytometry for mCherry and CD134 surface expression (Figure 4A). Transduction with a minimal trans-packaged lentiviral vector that encodes only mCherry and no viral proteins, was done in parallel as a control. Surface CD134 down-regulation was observed with both CT5-cherry and CT5-cherry^A+^. It was slightly greater with the latter. In contrast the vector encoding mCherry alone caused no CD134 down-regulation (Figure 4A). 

CrFK-CD134 cells were then transduced at equivalent MOIs with CT5efs and CT5efs^A+^ (Figure 4B). These two viruses do not carry reporter gene insertions but are Env (-) by virtue of a frameshift caused by a 29 nt insertion in *env* (Figure 1A). Viral protein expression was assessed by western blotting for FIV Gag/Pol expression in the target cells and this was equivalent for the two viruses (Figure 4C). Surface CD134 down regulation was observed with both viruses, but there was no significant difference between the OrfA (+) virus compared to the OrfA (-) virus, even at an MOI of 2.6 (90% of cells infected). These experiments suggest that viral determinants other than Env or OrfA contribute to down-regulation of CD134. 

## 3. Experimental Section

### 3.1 Cell lines.

Crandell feline kidney (CrFK) cells and FeT-J cells were obtained from the ATCC. Feline KE-R cells were kindly provided by C. Münk (Heinrich Heine University, Düsseldorf). G355-5, Mya-1, and murine L2.23 cells, which produce human IL-2 used for culture of PBMC and Mya-1 cells, were gifts from T. Miyazawa (Kyoto University, Japan). CrFK, KE-R and G355-S cells were maintained in Dulbecco’s modified Eagle medium (DMEM) with 10% heat-inactivated fetal calf serum (FCS), penicillin/streptomycin and L-glutamine. FeT-J and Mya-1 were maintained in RPMI with 10% FCS and penicillin-streptomycin and L-glutamine with additions for FeT-J (0.1% β-mercaptoethanol) and Mya-1 (20% FCS, 10% D-glucose, 1% sodium bicarbonate, 1% sodium pyruvate, IL-2, and 1% non-essential amino acids). Feline peripheral blood mononuclear cells (PBMC) were obtained by Ficoll centrifugation from whole blood and were cultured in RPMI medium supplemented with 10% FCS, 2 mM glutamine, 1 mM sodium pyruvate, essential and nonessential amino acids, 10 mM HEPES, 0.05 mM β-mercaptoethanol, phytohemagglutinin E (PHA-E; 2 µg/ml), and IL-2.

### 3.2 Generation of CrFK-CD134 cells.

A stable feline CD134-expressing cell line (CrFK-CD134) was derived by transducing CrFK cells with a pDONAI-derived gammaretroviral vector that encodes CD134 ([51]; kind gift of B. Willett, University of Glasgow). For this purpose VSV-G-pseudotyped retroviral vector particles were prepared by 293T cell transfection with Moloney murine leukemia virus packaging plasmid pHIT60 [52] and a VSV-G expression plasmid. The transduced cells were selected in 800µg/ml G418 and single cell clones were derived by limiting dilution. Cell surface expression was assessed before and after cloning by flow cytometry with anti-CD134 (MCA 2568A488T, AbD Serotec).

### 3.3 Construction of pCT5 based reporter vectors.

Overlap extension PCR was first used to repair the premature stop codon in the pCT5 *orfA* gene (an alternative name for this gene is *orf2*). Outer primers were GTTTTACCTCTTGAATTTCGTTCC and GATTGGCAGGTAAGTAGAAGACTC, and inner primers were CTATATCTCCAAAATAATCCCTGCAGTAATCTAATAGCTTTGTCCC and ATTACTGCAGGGATTATTTTGGAGATATAGATTTAAGAAACCC. This follows the strategy of Waters et al. [38] to change the *orfA* premature stop codon to TGG (Trp) (Figure 1A). This construct was called pCT5^A+^. We then exchanged the KpnI-BstB1 segment spanning *orfA* from pCT5^A+^ into pCT5efs; the latter is a version of pCT5 in which a short (29 nt) insertion in *env* generates a frameshift [53]. This yielded pCT5efs^A+^. Next, we introduced reporter genes encoding GFP and mCherry into the FIV envelope such that each was in frame with *env* and was preceded by porcine teschovirus 2A (P2A) peptide (ATNFSLLKQAGDVEENPG°P) [36]. pCT5 was digested with BglII and the smaller fragment was ligated and called pCT5deltaBglII_S_, the larger fragment (pCT5deltaBglII_L_) was treat with calf intestinal phosphatase and kept for use as a backbone for reinsertion. A P2A-*gfp* amplicon was synthesized by PCR using a pre-existing template (data not shown) with the primers sP2Agfp (TTTCCTAGGAGCCACGAACTTCTCTCTGTTAAAGC) and aP2Agfp (AAACTAGTTTACTTGTACAGCTCGTCCATGCCGAG) and was inserted into pCT5deltaBglII_s_ between AvrII and SpeI; this strategy results in replacement of 1.56 kb of *env* with the P2A-GFP insert but does not intrude upon any other reading frames or known cis-acting elements, such as either Rev exon or the RRE and was therefore predicted to not interfere with the Rev-dependent splicing program of FIV. This intermediate was then digested with BglII and ligated with PCT5deltaBglII_L _to generate the reporter virus pCT5-GFP. The segment between BlpI and KpnI was exchanged into pCT5^A+^ to obtain pCT5-GFP^A+^. pCT5-Cherry and pCT5-cherry^ A+^ were constructed analogously using primers sP2Acherry (TTCCTAGGAGCCACGAACTTCTCTCTGTTAAAGCAAGCAGGAGACGTGGAAGAAAACCCCGGtCCTATGGTGAGCAAGGGCGAGG) and aP2Acherrry (AAACTAGTTTACTTGTACAGCTCGTCC).

### 3.4 Single-round FIV reporters and full length replicating FIV production.

VSV-G pseudotyped reporter virus particles were produced by transfection of 293T cells as described [6,40]. Supernatants were harvested 48h later, filtered (0.45 µM), concentrated by ultracentrifugation over a sucrose cushion in a swinging bucket SW32Ti rotor at 25,000 rpm for 2 h, aliquoted and frozen at -80°C. Replication competent virus production was performed by 293T cell transfection of pCT5 and pCT5^A+^. Reverse transcriptase (RT) activities were determined using a ^32^P-based microtiter plate assay as described previously [6] and titration was performed on CrFK cells using a focal infectivity assay [32]. Mean RT activity +/- s.d. from duplicate measurements was calculated for each sample. To assess GFP and m-cherry expression, cells were fixed in 4% paraformaldehyde and analyzed on a FACScan (BD Biosciences).

### 3.5 Immunoblotting.

Equal number of cells uninfected or infected with either CT5efs or CT5efs^A+^ viruses at an MOI of 2.6 were lysed in RIPA buffer (150 mM NaCl, 0.5% deoxycholate, 0.1% sodium dodecyl sulfate, 1% NP-40, 150 mM Tris-HCl, pH 8.0) with protease inhibitors (Complete mini; Boehringer). For Figure 1B, cells were processed similarly after transfection with the reporter viruses or plasmids that express only GFP or mCherry under transcriptional control of the hCMV promoter. The lysates were boiled in Laemmli buffer with β-mercaptoethanol for 10 min, electrophoresed in 10% Tris-HCl gels (Bio-Rad), and transferred over one hour to a PVDF menbrane (Bio-Rad). The blocked membrane was incubated with serum from a cat infected with FIV PPR (kind gift of S. VandeWoude) or anti α-tubulin antibodies (Sigma) for one hour at 1:1000 dilution, then washed with Tris-buffered saline–Tween 20 (TBST) three times for 7 min each. Afterward, membranes were incubated for 1h at room temperature with the secondary goat anti-cat at 1:1000 (MP Biomedicals 55293) and goat anti-mouse horseradish peroxidase (Calbiochem), at 1:4000. After washing with TBST 3 times for 10 min each, membranes were incubated in SuperSignal West Pico chemiluminescent substrate (Pierce) for 1 to 2 min and exposed to film. For Figure 1B, the primary antibodies used were monoclonal mouse anti-GFP (Clonetch) and polyclonal rabbit anti-mCherry at 1:5000 for 1h; while the secondary antibodies were goat anti-mouse and anti-rabbit horseradish peroxidase (Calbiochem), at 1:4000 for 1h. 

## 4. Discussion and Conclusions

We describe a set of four FIV reporter viruses in which fluorescent proteins are expressed as 2A peptide fusions within Env. The reporters can be produced at high titer using standard transient transfection methods in the primate cell lines that are widely used for genetically-defined virus and reporter virus production. This is an advantage since there are no feline cell lines that are readily transfected and we have previously shown that the only block to FIV virion production in human cells is the inactive U3 element promoter [5,32]. Alternative marker gene arrangements may be feasible for FIV but the present insertion into *env* preserved viral production and infectivity.

These reporter viruses add to the experimental armamentarium by allowing the FIV life cycle to be assessed in the same manner as HIV-1 has been assessed for years [2,3,25]. To illustrate capacity for addressing a specific virological question, we initiated studies with OrfA (+) and (-) reporters. Besides Vif, for which a clear role in APOBEC3 antagonism has been identified [10,11,15], OrfA is the only other known FIV accessory protein. Its function or functions in the FIV life cycle are not clear at present. We confirm the prior reports [37,38,39] that this protein is needed for FIV to replicate in feline PBMC (Figure 1C) and show that it also enhances replication in feline T cell lines (Figure 1D,E). As Vif, Vpx, Nef and Vpu have been shown in the primate lentiviruses to function mainly to counter species-specific restriction factors [54], it is reasonable to conjecture that OrfA could be involved in such activities. Several reports have also described primate lentivirus Tat protein-analogous (LTR-transactivating) function [45,48], effects on cellular mRNA profiles [49], virus particle production [39] and a receptor (CD134) down-regulation function [46]. While this protein has so far received relatively limited study, lentiviral accessory protein multi-tasking would not be surprising. For example, HIV-1 Vpu mediates primary receptor (CD4) down-regulation [55] and also counteracts the restricting activity of BST-2/Tetherin [56]. Whether or not effects on CD134 are a main contributor to the OrfA requirement for PBMC replication is deserving of further study. To apply the reporter viruses to this phenomenon, we generated CD134 (+) and (-) cell lines and tested OrfA(+) and (-) reporter virus infection. Our results raise the possibility that CD134 down-regulation is not mainly a consequence of OrfA but may result from expression of other viral proteins. The reporter viruses will facilitate additional work in this area. We also did not detect a specific effect of OrfA on the viral production and assembly phase in transfected 293T cells (Figure 2). 

In summary, the present work makes available a set of FIV reporter viruses that can be used for single cycle analyses of the FIV life cycle while expressing all viral proteins but Env *in cis*. 
